# Biological features between miRNAs and their targets are unveiled from deep learning models

**DOI:** 10.1038/s41598-021-03215-w

**Published:** 2021-12-10

**Authors:** Tongjun Gu, Mingyi Xie, W. Brad Barbazuk, Ji-Hyun Lee

**Affiliations:** 1 Bioinformatics, Interdisciplinary Center for Biotechnology Research, University of Florida, Gainesville, FL USA; 2 Division of Quantitative Sciences, University of Florida Health Cancer Center, University of Florida, 2033 Mowry Road, Gainesville, FL 32610 USA; 3 University of Florida Health Cancer Center, University of Florida, Gainesville, FL USA; 4 Genetics Institute, University of Florida, Gainesville, FL USA; 5 Department of Biochemistry and Molecular Biology, University of Florida, Gainesville, FL 32610 USA; 6 Department of Biology, University of Florida, Gainesville, FL USA; 7 Department of Biostatistics, University of Florida, Gainesville, FL USA

**Keywords:** Computational biology and bioinformatics, Machine learning

## Abstract

MicroRNAs (miRNAs) are ~ 22 nucleotide ubiquitous gene regulators. They modulate a broad range of essential cellular processes linked to human health and diseases. Consequently, identifying miRNA targets and understanding how they function are critical for treating miRNA associated diseases. In our earlier work, a hybrid deep learning-based approach (miTAR) was developed for predicting miRNA targets. It performs substantially better than the existing methods. The approach integrates two major types of deep learning algorithms: convolutional neural networks (CNNs) and recurrent neural networks (RNNs). However, the features in miRNA:target interactions learned by miTAR have not been investigated. In the current study, we demonstrated that miTAR captures known features, including the involvement of seed region and the free energy, as well as multiple novel features, in the miRNA:target interactions. Interestingly, the CNN and RNN layers of the model perform differently at capturing the free energy feature: the units in RNN layer is more unique at capturing the feature but collectively the CNN layer is more efficient at capturing the feature. Although deep learning models are commonly thought “black-boxes”, our discoveries support that the biological features in miRNA:target can be unveiled from deep learning models, which will be beneficial to the understanding of the mechanisms in miRNA:target interactions.

## Introduction

miRNAs are small non-coding RNAs that have an average length of ~ 22 nucleotides (nts)^1^. They typically form base-pairs with their target RNAs within the RNA-induced silencing complex and act to repress gene expression post-transcriptionally^2^. It has been reported that miRNAs play key roles in a variety of biological processes and human diseases^3–5^, and several miRNA-targeted therapeutics have undergone clinical trials for treating human cancers^6–8^ Thus, it is important to identify the targets of the miRNAs to better understand the function and regulation of miRNAs.

Recently, we developed a hybrid deep learning-based approach to predict miRNA targets, named miTAR^9^. miTAR integrates two major deep learning (DL) algorithms, convolutional neural networks (CNNs) and recurrent neural networks (RNNs). CNNs are designed to learn spatial features and RNNs are designed to learn sequential features. Our approach has the advantages of learning both the intrinsic spatial and sequential features of miRNA:target interactions. We applied miTAR on two datasets, DeepMirTar and miRAW^10,11^; both contain a large number of widely used validated miRNA:targets pairs (positive pairs) and negative pairs (miRNAs and non-target pairs) (Details in the Methods). We obtained two models: miTAR1 trained on the DeepMirTar dataset; and miTAR2 trained on the miRAW dataset. We have demonstrated that miTAR has substantially improved performance relative to all current DL approaches^9^; however, the features miTAR learned have not been investigated, which is important for the understanding of the mechanisms in miRNA:targets interactions.

Multiple common features have been widely used in predicting miRNA targets. They are seed match, free energy, target site accessibility, and sequence conservation^12,13^. The seed region of a miRNA is thought to be important for miRNA to recognize its target. It is commonly defined as seven nucleotides starting from the second nucleotide of the miRNA^14^. There are multiple types of seed match, but the most effective miRNA targets include a perfect Watson–Crick match between the 2–7 nucleotide of miRNA and its target RNA/gene^3,12^. Thus, in the current study, we defined the seed region as the 2–7 nucleotide region of a miRNA. Free energy can be used to measure the binding between miRNAs and their targets and is widely used in miRNA target prediction. Free energy is a negative real value. The lower the value the stronger the binding, therefore, the target is more likely to be a true target of the miRNA with lower free energy^13^. Site accessibility is the measure of how accessible the target region for a miRNA binding is; sequence conservation is a measure of how conserved the target sequence is across species. They both are also widely used in many studies^12,13^.

DL has been successfully applied in many scientific fields; however, the interpretation of DL models is not well studied^15,16^. Interpretation of DL models is not only critical for developing robust and reliable new models but also important for understanding the underlying biological mechanisms. Thus, the interpretation of DL models has been reignited in recent years. However, among the recent developments, the majority were applied to image and natural language processing, such as gradient-based approaches, Grad-CAM++, Layer-wise Relevance Propagation, Local Interpretable Model-agnostic Explanations^16,17^. A few approaches have been applied to biological data. Zhou and Troyanskaya^18^ used in-silico mutagenesis analysis to evaluate the impact of genomic sequence features. Shrikumar et al.^19^ used deepLift, a Gradient-based backpropagation method, to discover transcription factor motifs. As far as we are aware, no methods have been applied to DL models on miRNA target interpretation. In this work we applied multiple approaches, including a series of in-silico mutagenesis analyses and correlation analyses, to demonstrate that our DL model can capture both known and novel features existing in miRNA:target interaction. Furthermore, we demonstrated that CNN and RNN perform differently on capturing features.

## Results

### In-silico mutagenesis analyses demonstrated that miRNA seed region significantly impacts miRNA:target interactions

**Figure 1:**
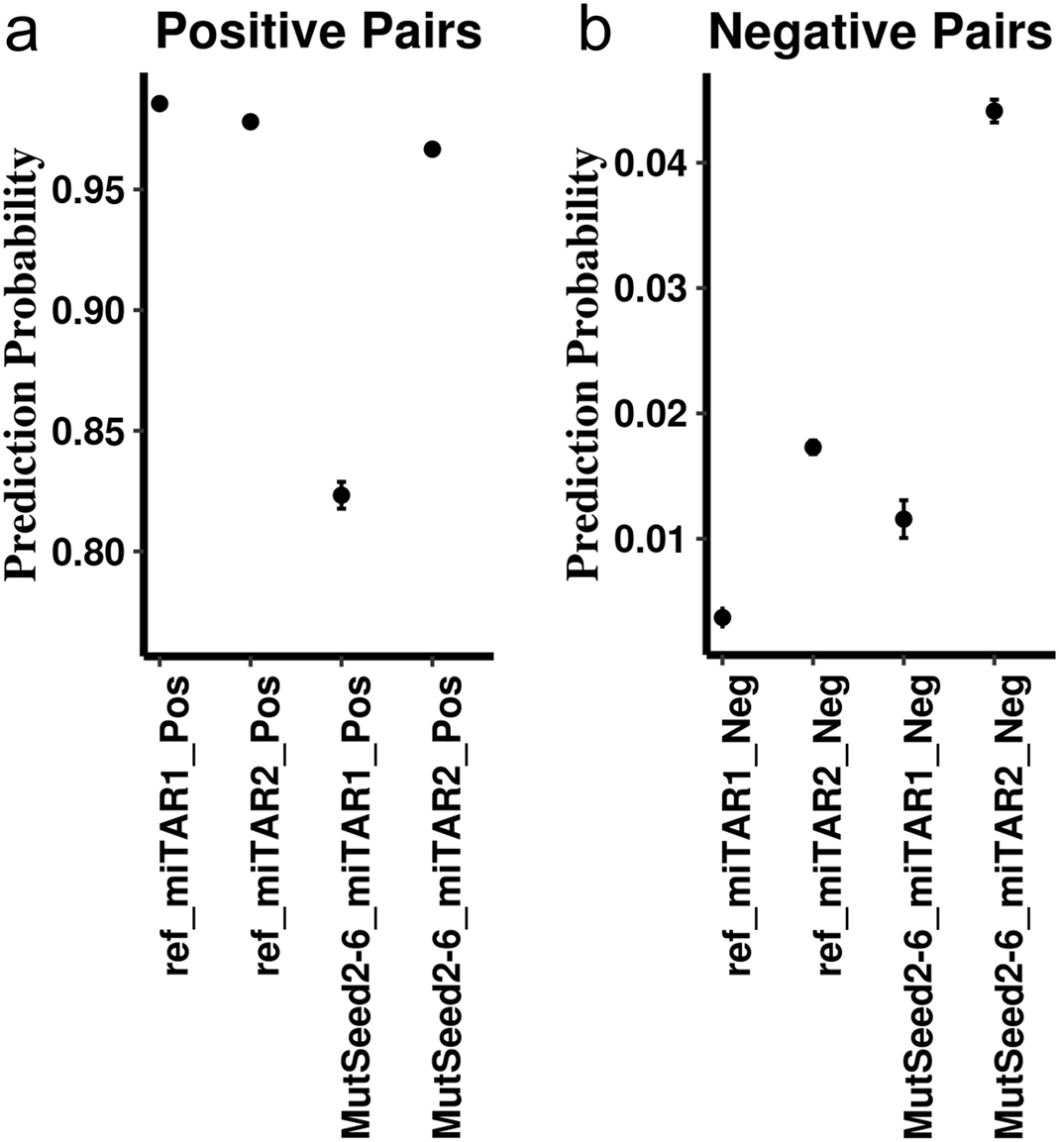
The impact of the alteration at the seed region for two models with the matched dataset (miTAR1 with DeepMirTar; miTAR2 with miRAW). Left figure (**a**) shows the alteration for the positive miRNA:target pairs from DeepMirTar and miRAW. Right figure (**b**) shows the alteration for the negative pairs from the same two datasets. Y axis is the average prediction probability with standard errors. Prediction probability represents the probability predicted by our models for a sequence being a target sequence of a miRNA. Ref_miTAR1_Pos and ref_miTAR2_Pos represent the prediction probability from the raw sequences. MutSeed2-6_miTAR1_Pos and MutSeed2-6_miTAR2_Pos represent the prediction probability from the altered sequence (seed region was mutated to ‘N’s).

We first examined whether the seed region plays an important role in the prediction. We defined the seed region as the six nucleotides from miRNA position 2nd to 7th in the miRNA, which is the least number of nucleotides forming perfect matches between miRNAs and their targets^12^. We performed in-silico mutagenesis analysis by changing the nucleotides of the seed region in miRNAs to ‘N’s and then compared the performance on predicting miRNA target between the wild-type miRNA sequences and the altered sequences. The seed region significantly alters the prediction probability on both DeepMirTar and miRAW positive and negative miRNA:target pairs (p-values < 0.05 after Bonferroni multiple testing correction). The alteration is much larger in DeepMirTar positive pairs and miRAW negative pairs (Fig. 1). In addition, we mutated the seed region to ‘G’s, which is the nucleotide with the least number at the seed region in the DeepMirTar and miRAW datasets. We obtained similar results (Supplementary Fig. 1). The consistent results between the two types of mutagenesis analysis suggest the seed region plays an important role in the interactions of miRNA:target for a large portion of the known miRNA:target pairs.

### CNN and RNN behave differently at capturing the free energy

CNN excels in learning spatial features and RNN discerns sequential features^20^. To evaluate the performance of CNN and RNN in our models, we calculated the free energy for each pair of miRNA:target. The free energy is less for miRNA:target pairs with more base-pairings, which can be considered a spatial feature. We performed Spearman’s correlation coefficient analysis between the free energy and the layer output of CNN and RNN. We first did the correlation analysis for the output of each CNN unit with the free energy using the DeepMirTar positive miRNA:target pairs. We found that the maximum (max) correlation is 0.32 and the minimum (min) correlation is − 0.34 (Table 1). Then we summed the unit outputs of CNN per each feature map and performed the correlation analysis between the summed feature map output and the free energy. The max and min correlations are increased: 0.51 for the max correlation and − 0.53 for the min correlation (Table 1). Furthermore, we summed the outputs for all the units of CNN and correlated it with the free energy. The max and min correlations are further increased: the max correlation is 0.59 and the min correlation is − 0.56 (Table 1). Similar results were obtained for the negative pairs in DeepMirTar, and miRAW positive and negative pairs (Table1 and Supplementary Table 1). The analysis results suggest CNN captures the spatial features in the interactions of miRNA:target and the features captured by the units within a feature map or across feature maps of CNN are partially overlapped.

**Table 1:** The maximum (Max) and minimum (Min) correlation coefficient (Cor) between the outputs of CNN and RNN layer with the highest free energy for the positive pairs from DeepMirTar and miRAW.

	UnitCount	MinCor	MinCor_Pval	MaxCor	MaxCor_Pval
**DeepMirTar CNN positive vs the highest binding energy**					
Raw	21,438	− 0.34	1.85E−104	0.32	2.78E−96
CombinePerFeatureMap	640	− 0.53	5.93E−287	0.51	1.21E−255
CombineAllUnits	2	− 0.56	0	0.59	0
**miRAW CNN positive vs the highest binding energy**					
Raw	17,486	− 0.19	2.77E−32	0.19	3.19E−31
CombinePerFeatureMap	632	− 0.46	2.26E−209	0.40	7.31E−152
CombineAllUnits	2	− 0.61	0	0.57	0
**DeepMirTar BiRNN positive vs the highest binding energy**					
Raw	58	− 0.19	4.17E−33	0.36	4.16E−117
Combine_AllUnits	2	− 0.10	1.27E−08	0.22	1.82E−44
**miRAW BiRNN positive vs the highest binding energy**					
Raw	64	− 0.30	4.23E−81	0.27	1.89E−66
Combine_AllUnits	2	− 0.14	8.95E−18	0.20	1.26E−36

*The p-values (Pval) are from Spearman's correlation coefficient analysis testing the coefficient equals to zero and adjusted for the multiple comparisons.

We conducted a similar analysis for the RNN layer. First, we did Spearman’s correlation coefficient analysis between each unit output and the free energy. The max (0.36) correlation is close to the CNN max unit correlations and the absolute min (− 0.19) correlation is smaller than the CNN min unit correlation for the DeepMirTar positive pairs; while the max (0.22) and min (− 0.30) correlations for the miRAW positive pairs are larger for the RNN layer than the CNN layer (max: 0.19 and min: − 0.19). These results indicate that RNN also captures the spatial features. However, when we summed the unit outputs of RNN, the max and min correlations for the summed output of RNN are largely reduced for both datasets and also for the negative pairs from both datasets (Table 1 and Supplementary Table 1). The results imply the free energy feature is captured independently across units of RNN. Therefore, collectively the CNN layer is better at capturing the spatial features than the RNN layer.

### Features identified from in-silico mutagenesis analyses from the DeepMirTar dataset

Both the seed region and the free energy are common features in miRNA:target interactions, which are captured by our models as demonstrated in the previous sections. In addition to the analysis of the known features, we explored whether we could identify novel features using our models from the two datasets (DeepMirTar and miRAW). We did six types of nucleotide in-silico mutagenesis analysis on the positive pairs of DeepMirTar and miRAW: one-, two-, three-, four-, five-, and six-nucleotide mutation. We measure how the mutation alters the prediction output compared to that of the wild-type sequence input. The larger the alteration in prediction output, the more likely that nucleotide is more important to the miRNA:target interactions. For multi-nucleotide mutagenesis analyses, the step between each mutation is one nucleotide. The miRNA positions were labeled with the letter, L, and the target RNA positions were labeled with the letters, LT.

**Figure 2:**
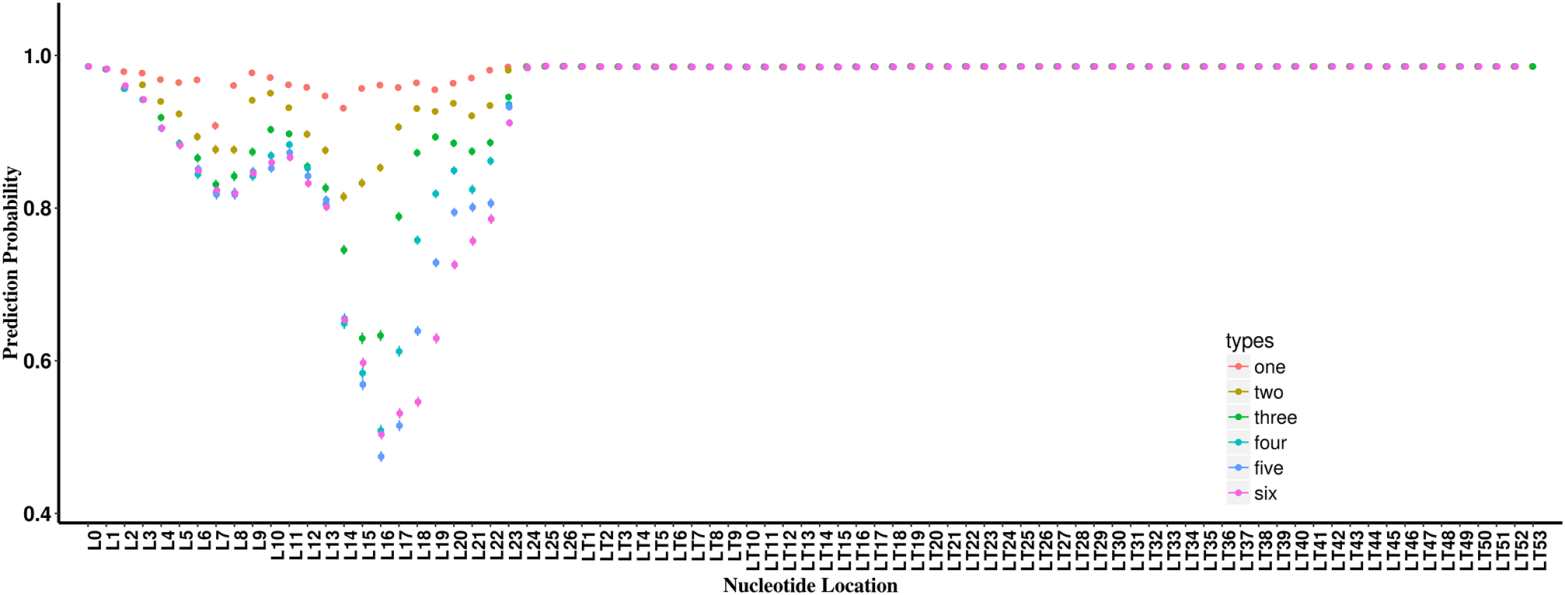
The prediction probability for the DeepMirTar positive pairs for six types of in-silico mutagenesis analyses: the one-, two-, three-, four-, five-, and six-nucleotide mutation. The miRNA positions were labeled with the letter, L, and the target RNA positions were labeled with the letter, LT. For the multi-nucleotide mutation, the prediction probability was recorded at the stop position of the mutation. miRNA nucleotides were ordered from 5′→3′ and the target nucleotides were ordered from 3′→5′. L0 is the prediction probability of the raw sequence.

For the DeepMirTar positive pairs, two peaks were found within the distribution for all six mutagenesis analyses (Fig. 2). In the one-nucleotide mutation examination, one peak was found in the vicinity of miRNA nucleotide 7 (labeled miTAR1_L7) and one peak was found near nucleotide 14 (labeled miTAR1_L14). With more nucleotides being mutated, the two peaks become deeper, and the summit of the miTAR1_L14 peak is shifted towards L16 while the summit of the miTAR1_L7 peak shifts between L7 and L8. Because the nucleotides in the multi-nucleotide mutagenesis analyses were mutated from larger positions to smaller positions (details in “Methods”), the nearby nucleotides at smaller positions were still included in the mutagenesis analysis. Nevertheless, the changes of the peaks suggest the nearby nucleotides are important. Notably, the five-nucleotide mutation reaches the largest alteration on the prediction probability. The alteration is larger than 50% at the summit for the miTAR1_L14 peak of the five-nucleotide mutagenesis analysis suggesting that the mutation alters the prediction from being a target of miRNA to a non-target. This further supports the importance of the nearby nucleotides. The six-nucleotide mutation also generates strong alterations but less than the five-nucleotide mutation, suggesting more nucleotide alterations beyond five continuous nucleotides may not greatly affect the miRNA:target interactions.

**Figure 3:**
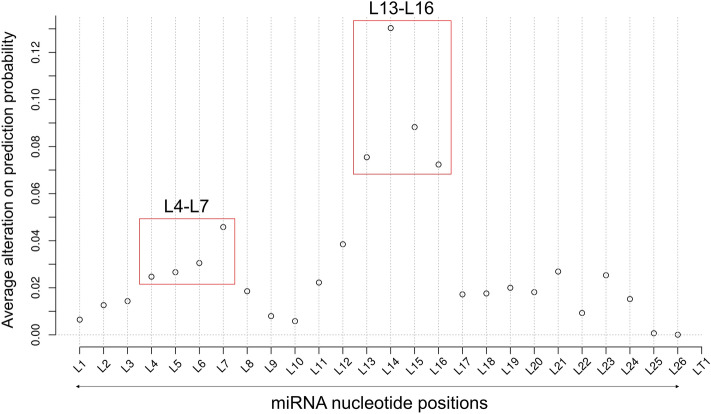
Average nucleotide alterations on prediction probability for the DeepMirTar positive pairs. L marks the position of miRNA nucleotides and LT marks the position of target nucleotides.

To determine the impact of the single nucleotide from the six types of multi-nucleotide mutagenesis analysis, we summed the impact of the single nucleotide mutation and multi-nucleotide mutation. For the multi-nucleotide mutation, the single nucleotide impact was calculated from two adjacent types of multi-nucleotide mutation. An example was shown in Supplementary Fig. 2. In the end, we added all the single nucleotide impacts, and the average value was used to represent the overall importance of a single nucleotide (details in “Methods”). The results are shown in Fig. 3 for the DeepMirTar dataset. Consistent with the single nucleotide mutagenesis analysis, miTAR1_L7 and miTAR1_L14 show the strongest impact in the two peaks. In the first peak, nucleotides L4 to L7 show stronger impacts than others. Thus, these four nucleotides were selected as a feature and labeled miTAR1_L4-4. In the second peak, nucleotides L13 to L16 show stronger impacts than others. Similarly, these four nucleotides were selected as another feature and labeled miTAR1_L13-4.

### Features identified from in-silico mutagenesis analyses from the miRAW dataset

**Figure 4:**
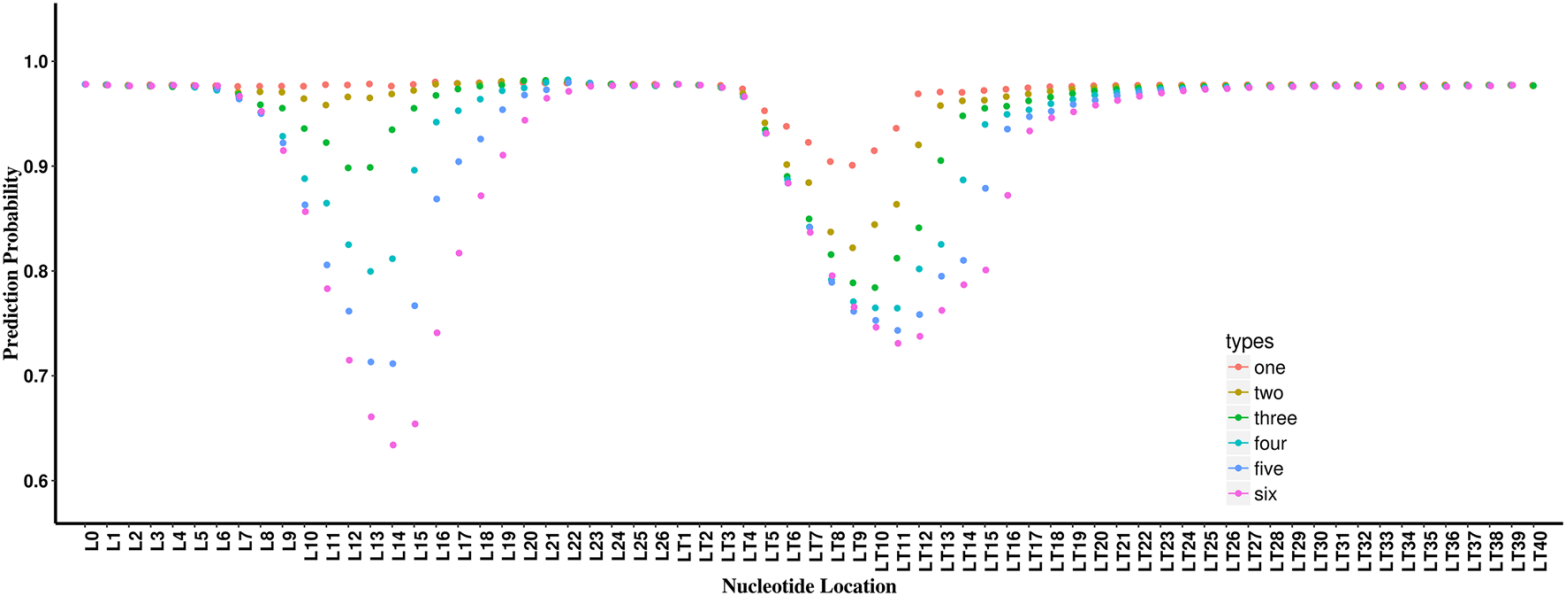
The prediction probability for the miRAW positive pairs for the one-, two-, three-, four-, five-, and six-nucleotide mutation. The labels have the same meaning as shown in Fig. 2.

We performed analyses on the miRAW dataset similar to those performed on the DeepMirTar dataset. For the miRAW positive pairs, at one-nucleotide mutation, one peak appears around the target RNA location LT9. In the multi-nucleotide mutagenesis analyses, a second peak appears (labeled L11, the first summit in two-nucleotide mutagenesis analysis), and it becomes stronger than the LT9 peak with an increased number of mutant nucleotides (Fig. 4). With more nucleotides being mutated, the two peaks become deeper and the summits for both peaks are shifted toward larger positions: the summit for the LT9 peak shifts to LT11; the summit for the L11 peak shifts to L14. Similar to the analysis for the DeepMirTar positive pairs, the changes of the two peaks indicate the nearby nucleotides are important for miRNA:target interactions. We also observed that from three-nucleotide mutations, the growth of the LT9 peak slows down. We did seven- and eight-nucleotide mutagenesis analyses specifically for the miRAW positive pairs. The LT9 peak reaches its maximum alteration on prediction output at seven-nucleotide mutations, which is almost the same as the six-nucleotide mutation (Supplementary Fig. 3). Although the L11 peak does not reach its maximum alteration, the increase on the summit slows down (Supplementary Fig. 3). The results also support that, similar to the DeepMirTar dataset, miRNA:target interactions do not benefit greatly beyond six continuous nucleotides.

**Figure 5:**
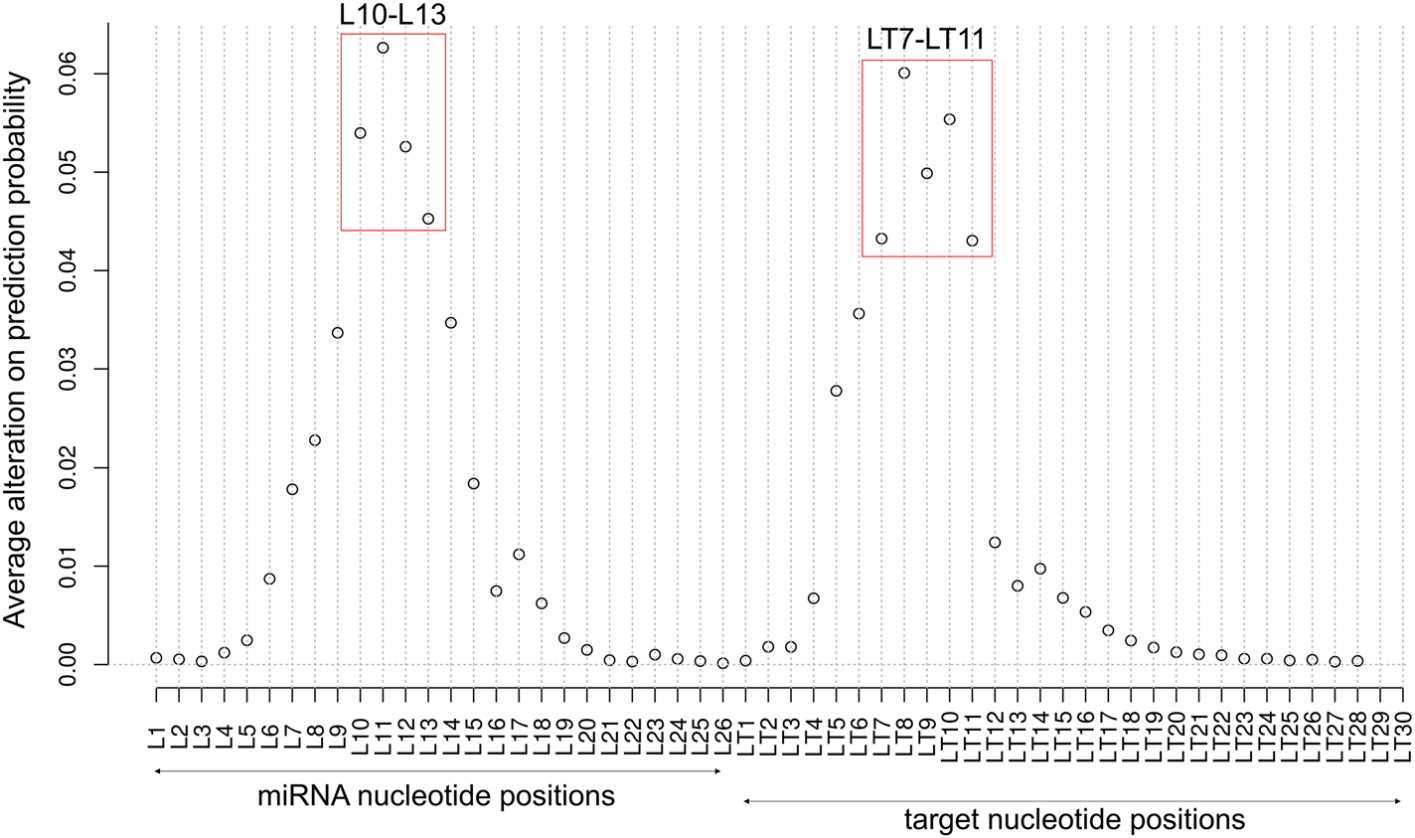
Average nucleotide alterations on prediction probability for the miRAW positive pairs. L marks the position of miRNA nucleotides and LT marks the position of target nucleotides.

To obtain the single nucleotide impacts from miRAW in a similar fashion to the analysis of the DeepMirTar dataset*,* we accumulated the impacts with or without surrounding nucleotides from one-nucleotide mutagenesis analysis and two adjacent types of mutagenesis analyses. The results are shown in Fig. 5. For the LT9 peak, nucleotides 7 to 11 show stronger impacts than others and were selected as one feature, labeled miTAR2_LT7-5. Because the target sequences were ordered from 3′→5′ and the miRAW dataset extended five nucleotides on both ends of the target site, miTAR2_LT7-5 locates towards the 3′ end of the target that most likely matches the 5′ end of miRNA by complementary base-pairing. Thus, miTAR2_LT7-5 potentially base pairs with the seed region of miRNAs. For the L11 peak, nucleotides 10 to 13 show stronger impacts than others, and were selected as another feature and labeled miTAR2_L10-4.

### The independent and joint effects of the identified features

**Figure 6:**
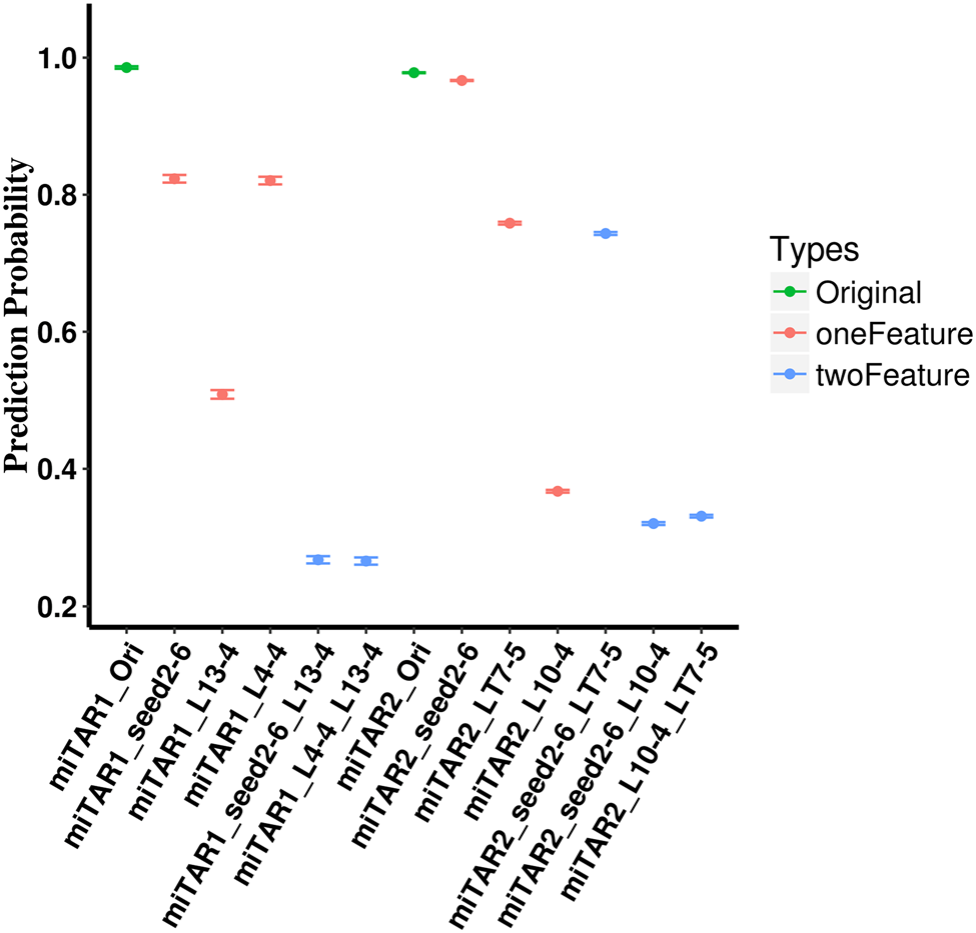
The joint and independent impacts of two types of features identified from the in-silico mutagenesis analyses. All the analyses were done using the data from positive pairs of DeepMirTar (the first six columns with the label of miTAR1) and miRAW (the remaining columns with the label of miTAR2). The Y axis is the average prediction probability with standard errors. Ori marks the prediction probability for the raw sequences. seed2-6 marks the prediction probability after altering the seed region. L13-4 marks the prediction probability after altering the region from L13 to L16. L4-4 marks the prediction probability after altering the region from L4 to L7. LT7-5 marks the prediction probability after altering the region from LT7 to LT11. L10-4 marks the prediction probability after altering the region from L10 to L13.

We first evaluated the independent and joint effects of three regions: the seed region, and two regions identified from DeepMirTar dataset (miTAR1_L4-4 and miTAR1_L13-4). We mutated the three regions separately to obtain the independent effects and two of the three regions to obtain the joint effects. The results were shown in Fig. 6. The seed region (labeled as seed2-6) and miTAR1_L4-4 show similar effects on the prediction probability (~ 0.16 reduction on prediction probability for both regions) while miTAR1_L13-4 has a larger effect (~ 0.48 reduction on prediction probability). Because the miTAR1_L4-4 is part of the seed region, and the similarity observed for the DeepMirTar dataset, miTAR1_L4-4 is most likely the key element of the seed region. The same conclusion is also supported by the joint effects: the joint effects of seed region and miTAR1_L13-4 are similar to the joint effects of miTAR1_L4-4 and miTAR1_L13-4 (~ 0.72 reductions). However, the joint effects reduced the prediction probability at a relatively larger scale than the sum of the independent effects from the miTAR1_L4-4 and miTAR1_L13-4 region (0.72 > 0.48 + 0.16).

We conducted a similar analysis for the seed region, miTAR2_LT7-5 and miTAR2_L10-4 (Fig. 6) in the miRAW dataset. The seed region generates a small but statistically significant effect (~ 0.01 reduction on prediction probability; p-value = 0.0 from Mann–Whitney *U* test). The miTAR2_LT7-5 shows a larger effect with ~ 0.22 reduction on prediction probability, while miTAR2_L10-4 generates the largest independent effect (~ 0.61 reductions) suggesting the L10-4 region is potentially the biggest factor for the miRAW dataset. The joint effect of the seed region and miTAR2_L10-4 (~ 0.66 reductions), and the joint effect of the miTAR2_L10-4 and miTAR2_LT7-5 (~ 0.65 reductions) show similar impacts on prediction probability. The joint effect from the seed region with the miTAR2_LT7-5 generates the smallest impact (0.23 reduction), which is similar to the independent effect of miTAR2_LT7-5. The joint effect analyses for the miRAW dataset imply that miTAR2_LT7-5 potentially replaces the role of the seed region in miRNA:target interactions. Nevertheless, the joint effects with the seed region from the DeepMirTar and miRAW datasets suggest that the seed region plays a fundamental role in the miRNA:target interactions, while the other features boost the impact of the seed region.

### Base-pairing pattern in the identified features

It is well known that the base-pairing is extensive within the seed region of canonical miRNA targets. To examine whether the newly discovered features share the same level of base-pairing as the seed region, we used miRanda to predict the alignment between miRNA and its matched target. Then we counted how many base-pairings appeared at the feature regions. We first examined the 3,958 DeepMirTar positive miRNA:target pairs for the three regions: the seed region, miTAR1_L4-4, and miTAR1_L13-4 (Table 2). The seed region has an average of ~ 89.02% base-pairing, which is much higher than the base-pairing at miTAR1_L13-4 (~ 66.57%). Surprisingly, the miTAR1_L4-4 portion of the seed region has higher base-pairing (~ 94.76%) than the entire seed region which suggests that miTAR1_L4-4 may be the core of the seed region. We also examined miTAR1_L7 and miTAR1_L14. The base-pairing at miTAR1_L7 (~ 92.90%) lies in between miTAR1_L4-4 and the seed region; miTAR1_L14 (~ 68.12%) has higher base-pairing than the miTAR1_L13-4. We further extracted the miRNA:target positive pairs that alter the prediction probability > 0.48 (the average alteration on prediction probability) for miTAR1_L13-4. We obtained relatively higher base-pairing for the extracted pairs than all pairs at the seed region, while relatively lower base-pairing at miTAR1_L13-4 region. The results indicate that the base-pairings at miRNA 13 to 16 are much lower than the seed region, but that alterations to these base-pairings potentially impact the binding between miRNAs and their targets.

**Table 2:** The percentage of the base pairing for the features identified from DeepMirTar and miRAW datasets.

	Features identified from DeepMirTar dataset				Seed region	Features identified from miRAW dataset	
	L7	L14	L4-4	L13-4	L2-6	L10-4	LT7-5
**deepMirTar**							
All pairs	92.90%	68.12%	94.76%	66.57%	89.02%	70.55%	54.47%
Extracted_L13-4	91.48%	66.26%	94.53%	64.73%	90.01%	69.59%	54.35%
**miRAW**							
All pairs	95.39%	59.53%	96.07%	58.48%	94.46%	56.36%	86.49%
Extracted_L10-4	95.85%	57.44%	96.18%	55.64%	94.75%	53.56%	87.56%
Extracted_LT7-5	97.30%	63.25%	97.76%	60.67%	96.51%	56.48%	90.40%

We did the same analysis for the 32,661 miRAW positive pairs at the seed region, miTAR2_L10-4 and miTAR2_LT7-5 (Table 2). The base-pairing at miTAR2_L10-4 is much lower than at miTAR2_LT7-5 (56.4% vs 86.5%). The base-pairing at miTAR2_LT7-5 is comparable to the seed region (86.5% vs 94.5%), suggesting that miTAR2_LT7-5 plays a similar role as the seed region. We also extracted the miRAW positive pairs that alter the prediction probability > 0.61 by miTAR2_L10-4 and the positive pairs that alter the prediction probability > 0.23 by miTAR2_LT7-5 (0.61 and 0.23 are the average alteration on prediction probability for the respective features). We observed lower base-pairing for the extracted pairs than all pairs at miTAR2_L10-4. Compared to the higher base-pairing at miTAR2_LT7-5 for the extracted pairs versus all pairs, the results suggest that despite the base-pairing probability at miRNA 10–13 being much lower, it is important for the miRNA:target interactions.

We also compared all the regions across the two datasets (Table 2). Base-pairing is the highest at miTAR1_L4-4 for all the datasets, followed by miTAR1_L7, and then the seed region. The results further support that miTAR1_L4-4 is very possibly the core element of the seed region. The base-pairing at miTAR1_L14 is higher than miTAR1_L13-4 for DeepMirTar dataset, and both regions have higher base-pairing for DeepMirTar than miRAW indicating that miTAR1_L13-4 and miTAR1_L14 may be more important for the pairs in DeepMirTar than miRAW. The base-pairing at miTAR2_L10-4 for miRAW is the lowest for all the datasets. The base-pairing at miTAR2_LT7-5 is much higher for miRAW than DeepMirTar, and it is comparable to the base-pairing at the seed region for DeepMirTar. This further supports that miTAR2_LT7-5 potentially replaces the role of the seed region for the pairs in miRAW.

## Methods

### Datasets and models

The DeepMirTar and miRAW datasets were collected from two studies^10,11^ in our earlier work and the details are in^9^. Here is a brief description. DeepMirTar contains 3,908 positive miRNA:target pairs and 3,898 negative miRNA:target pairs. The positive pairs were originally obtained from three resources: miRecords, miRTarBase, and CLASH data. And only the target sites located in 3′UTRs with canonical seeds and non-canonical seeds were included in DeepMirTar^10^. The negative pairs were also obtained directly from the study of DeepMirTar^10^. First, they shuffled the canonical and non-canonical seed region of the real mature miRNAs until the region does not match the same region of all the real mature miRNAs. Then, a target region that matches the canonical and non-canonical seed region requirement for these shuffled miRNAs were obtained using miRanda^21^. These pairs were taken as negative pairs of the DeepMirTar dataset.

miRAW contains 32,660 positive and 31,993 negative pairs that were collected from two resources: Diana TarBase and MirTarBase. The positive target site sequences were obtained by cross-referencing with PAR-CLIP, CLASH, and TargetScanHuman 7.1. It contains many non-canonical targets. The negative target site sequences were obtained from the experimental validated non-target genes using two criterions: a maximum length of 30 nts and form a stable base-pairing. All the target and non-target site sequences were trimmed to the same length at 30 nts and further extended five nts on both sides. Thus, the length of the target sequences for miRAW is 40 nts. The length of the target sequences for DeepMirTar is highly variable, therefore, the target sequences were padded to the length of 53 nts, which is the longest target sequence of DeepMirTar. All the miRNA sequences from DeepMirTar and miRAW were padded to the length of the longest miRNA, 26 nts. In our previous work, we concatenated the sequence of a miRNA from 3′ to 5′ with its target sequence from 5′ to 3′. In our current work, we presented the results for miRNA sequences in the order of 5′ to 3′, and target sequences in the order of 3′ to 5′.

The miTAR model has six layers, including one embedding, CNN, Max pooling, and BiRNN layer, and two dense layers (details are in^9^). The inputs for the miTAR are the concatenated sequences of miRNAs and their matched target sites. The model trained on DeepMirTar was labeled miTAR1, and the model trained on miRAW was labeled miTAR2.

### The processes for in-silico mutagenesis experiments

We performed multiple types of mutagenesis analysis, including seed region mutation to evaluate and validate the known features and one- to six-nucleotide mutation to discover novel features. For each type of mutation, we altered the testing region/nucleotides in the input sequences to ‘N’/‘N’s and ran the prediction using the appropriate model on the matched dataset: we ran the miTAR1 model on DeepMirTar dataset; and miTAR2 on the miRAW dataset. The prediction probability (the probability predicted by our models for a sequence being a target site of a miRNA) was recorded for the original sequence and the altered sequence. The prediction probability from the original sequence was taken as the control to measure the effect of the mutation. The statistical analysis for the alteration on prediction between the wild-type sequence and the mutated sequence was carried out using the Mann–Whitney *U* test. Multiple testing correction was performed using Bonferroni correction. The threshold was set at a p-value of 0.05 after the Bonferroni correction.

For multi-nucleotides mutagenesis analysis, the step for each mutation was one nucleotide. The alteration at the edge of the wild-type sequences was done by padding zero. The location for each multi-nucleotide mutation was recorded at the stop location of the mutation. For example, the L3, L4, and L5 nucleotides were mutated for three-nucleotide mutation recorded at miRNA location L5.

### Free energy and base-pairing calculation

The base-pairing was obtained by aligning each pair of miRNA:target using miRanda (v1.9)^21^ with default settings except -sc 0 -en 0. The highest binding energy reported by miRanda for each pair was used for the correlation analysis with each of the CNN and RNN outputs. Using the function of *Spearmanr* from Python SciPy library, Spearman correlation coefficient and the corresponding p-value were calculated. The base-pairing in specific regions between miRNAs and their targets were counted by a customer script. The T:G/U:G was taken as one base pairing.

### Accumulative in-silico mutagenesis analysis

The impact of the nucleotide in a multi-nucleotide environment was evaluated using the following steps. Firstly, we performed the mutation analysis in the order from one- to six-nucleotide. Secondly, we calculated the impact of a nucleotide in the multi-nucleotide environment as the difference between the adjacent multi-nucleotide mutations. For example, the impact of the miRNA nucleotide 7 (L7) in the vicinity of three nucleotides on the left can be obtained by the differences between the three-nucleotide mutation at L6 and four-nucleotide mutation at L7 (formula (1); Supplementary Fig. 2); and the impact in the vicinity of three nucleotides on the right can be obtained by the differences between the three-nucleotide mutation at L10 and four-nucleotide mutation at L10 (formula (2); Supplementary Fig. 2). Thirdly, we summed the differences from every two adjacent types of mutagenesis and used the average value to demonstrate the impacts of the nucleotide [formula (3)].

1 $$e_{L7}^{3l} = e_{L7}^{4} - e_{L6}^{3} ,$$

2 $$e_{L7}^{3r} = e_{L10}^{4} - e_{L10}^{3} ,$$

3 $$e_{L7}^{3} = (e_{L7}^{3l} - e_{L7}^{3r} )/2,$$

where *e* represents the prediction probability difference between the mutated sequence and the original sequence; super-script 3 and 4 represent the number of nucleotides mutated; L6, L7, and L10 represent the miRNA nucleotide 6, 7, and 10 respectively; and *l* and *r* represent left and right.

### Figure plots

We used the R package, *ggplot2*, to plot Figs. 1, 2, 4, 6, Supplementary Fig. 1, and Supplementary Fig. 3.

## Discussion

DL models are normally “black boxes” to many users. For example, what features a DL model prioritized are hard to interpret. In our current work, we explore what we learned from our previously developed DL models^7^. Mutation and correlation analyses demonstrate that the models learned at least two known features: the seed region and free energy. Furthermore, we identified that CNN and RNN behave differently in learning the free energy feature. In the end, we performed mutagenesis analysis and identified multiple known and novel core elements that most likely play important roles in the miRNA:target interactions.

For DeepMirTar dataset, two regions were identified with the highest impact on the prediction probability: miTAR1_L4-4 with summit L7 and miTAR1_L13-4 with summit at L14. The miTAR1_L4-4 region overlapped with the seed region and has almost the same impacts on the prediction probability from independent and joint mutagenesis analyses (Fig. 6). Further, the miTAR1_L4-4 has the highest base-pairing, even higher than that of the seed region (Table 2). The seed region is thought to be an important element for miRNA recognition of their targets and is widely used in miRNA target prediction^3,12^. Our analysis suggests that four of the six nucleotides may define the core element of the seed region, and L7 is the most important site within the seed region. miTAR1_L13-4 is a reported feature that impacts the efficiency of miRNA’s function^3^, which is consistent with our result. We further identified that L14 is the most important site in miTAR1_L13-4 region. The joint effect of the miTAR1_L4-4 and miTAR1_L13-4 is similar to the joint effects of miTAR1_L13-4 and the seed region, which is higher than the sum of independent effects of miTAR1_L4-4 and miTAR1_L13-4 (Fig. 6). Similar observations were obtained with the miRAW dataset. The results indicate the seed region may serve as the basis to nucleate the interactions between miRNAs and their targets.

The two regions (miTAR2_L10-4 and miTAR2_LT7-5) identified from the miRAW dataset were not reported in other studies. Because miTAR2_LT7-5 locates towards the 3′ end of the target that most likely matches the 5′ end of miRNA by complementary base-pairing, and the seed region only minorly impacts the prediction probability in miRAW, it is possible that miTAR2_LT7-5 replaces the role of the seed region for the miRNAs in miRAW. This is further supported by the independent and joint mutagenesis analysis results (Fig. 6) and base-pairing analysis (Table 2). miTAR2_L10-4 locates at the 3′ side of the seed region in miRNAs. It is reported that the base-pairing beyond the seed region (towards the 3′end) defines the specificity for both canonical and non-canonical miRNA targets and is potentially essential for non-canonical miRNA targets^22^. Since miRAW contains both a large number of canonical and non-canonical miRNA targets^10^, their function most likely would be impacted by the two regions. Currently, no DL studies perform analyses from the perspective of target sequences. We demonstrate that the RNA/gene sequence (LT7-5) may contribute to the interaction with miRNAs. One possible reason may reflect evolutionary selection—under selection pressure, genes may increase nucleotide diversity to favor or impair miRNA binding.

In this study, we demonstrated using spearman’s correlation coefficient analysis that the RNN units is more unique than the CNN units on capturing the free energy feature in both DeepMirTar and miRAW datasets. One possible reason may be the order of RNN and CNN in miTAR. In miTAR, the RNN layer is behind the CNN layer. In order to determine whether and how large the order of the layers impact the performance of the layers, we performed the same correlation analysis for the Dense layer that is behind the RNN layer. Interestingly, we observed that most of the minimum and maximum correlations are not statistically significant (p-value > 0.05) (Supplementary Table 1). Of these significant correlations, there are no apparent changes between the unit and combined correlations (the changes of the correlation coefficients are within 0.01). The analysis results indicate in addition to the order of the CNN and RNN, the nature of CNN and RNN may play a role to explain the behavior differences observed in the correlation analyses.

miRNAs have been reported in many studies that are involved in many diseases, and various methods have been developed to predict miRNA-disease association^23–25^. In our earlier work, we demonstrated that miTAR performs substantially better than other alternatives. In the current work, we identified novel features in miRNA:target interactions. With a better approach and understanding of miRNA:target interactions, our work can contribute to the prediction of miRNA-disease association. We plan to integrate other omics data, for example gene expression, from a specific disease tissue to give a quantitative measurement of the impacts of miRNAs on their targets in the specific disease. These measurements can be used to improve the miRNA functional similarity used in miRNA-disease association analysis. Further, a network can be built between miRNAs and their target genes to explain the underling mechanism on the development of the disease.

In summary, using multiple approaches, we revealed multiple known and novel features from DL models that contribute to the interaction between miRNAs and their targets. Our analysis will not only benefit the understanding of the mechanism in miRNA’s functional process but also supplies a conceptual framework for unveiling the DL models.

## Supplementary Information

Supplementary Figures.

Supplementary Tables.
