# Supplementary material for: Biological features between miRNAs and their targets are unveiled from deep learning models
Source: Sci Rep. 2021 Dec 10;11:23825. doi: 10.1038/s41598-021-03215-w (PMC8664955; doi:10.1038/s41598-021-03215-w)
Supplement: Supplementary file 1 — Supplementary Figures. [file 41598_2021_3215_MOESM1_ESM.pdf]

Biological features between miRNA and their targets are unveiled from deep learning models

Tongjun Gu[1,2*], Mingyi Xie[3,4,5], W. Brad Barbazuk[1,4,6], Ji-Hyun Lee[2,7*]

[1]Bioinformatics, Interdisciplinary Center for Biotechnology Research, University of Florida, Gainesville, FL, USA
[2]Division of Quantitative Sciences, University of Florida Health Cancer Center, University of Florida, Gainesville, FL, USA
[3]University of Florida Health Cancer Center, University of Florida, Gainesville, FL, USA
[4]Genetics Institute, University of Florida, Gainesville, FL, USA
[5]Department of Biochemistry and Molecular Biology, University of Florida, Gainesville, FL 32610, USA
[6]Department of Biology, University of Florida, Gainesville, FL, USA
[7]Department of Biostatistics, University of Florida, Gainesville, FL, USA

Co-correspondence:
Tongjun Gu, Ph.D.
2033 Mowry Road, Gainesville, FL 32610
Phone: (352) 273-8058
Fax: (352)273-8070
Email: tgu@ufl.edu

Ji-Hyun Lee, DrPH
2033 Mowry Road, Gainesville, FL 32610
Phone: 352-273-9079
Email: jihyun.lee@ufl.edu

Legends for supplementary figures

Supplementary Figure 1. The impact of the alteration at the seed region for two models with the matched dataset (miTAR1 with DeepMirTar; miTAR2 with miRAW). The seed region was mutated to ‘G’s, which is the nucleotide with the least number at the seed region. Left figure (a) shows the alteration for the positive miRNA:target pairs from DeepMirTar and miRAW. Right figure (b) shows the alteration for the negative pairs from the same two datasets. Y axis is the average prediction probability with standard errors. Prediction probability represents the probability predicted by our models for a sequence being a target sequence of a miRNA. Ref_miTAR1_Pos and ref_miTAR2_Pos represent the prediction probability from the raw sequences. MutSeed2-6_miTAR1_Pos and MutSeed2-6_miTAR2_Pos represent the prediction probability from the altered sequence.

Supplementary Figure 2. An example of the calculation for the impact of the miRNA 7th nucleotide A in a three-nucleotide environment. A7 represents the 7th nucleotide A. X represents nucleotides that surrounds A7. The number following X represents the position of the nucleotide in a miRNA. $P_{X_4X_5X_6A_7}$ represents the the prediction probability for the altered sequence at miRNA position 4, 5, 6 and 7. $P_{X_4X_5X_6}$ represents the prediction probability for the altered sequence at miRNA position 4, 5 and 6. Similar meaning for the $P_{A_7X_8X_9X_{10}}$ and $P_{X_8X_9X_{10}}$.

Supplementary Figure 3. The prediction probability for the miRAW positive pairs for eight types of in-silico mutagenesis analyses: one-, two-, three-, four-, five-, six-, seven-, and eight-nucleotide mutation. The labels have the same meaning as shown in Fig. 2.

Legends for supplementary tables

Supplementary Table 1. The maxiamum (Max) and minimum (Min) correlation coefficient (Cor) between the output of CNN and RNN layer with the highest free energy predicted by miranda for the negative pairs from DeepMirTar and miRAW.

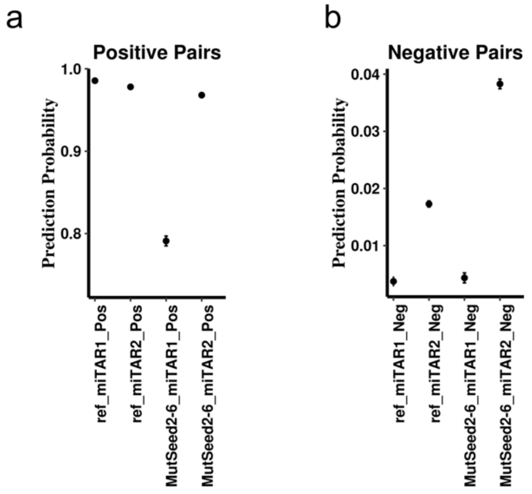

Supplementary Figure 1. The impact of the alteration at the seed region for two models with the matched dataset (miTAR1 with DeepMirTar; miTAR2 with miRAW). The seed region was mutated to 'G's, which is the nucleotide with the least number at the seed region. Left figure (a) shows the alteration for the positive miRNA:target pairs from DeepMirTar and miRAW. Right figure (b) shows the alteration for the negative pairs from the same two datasets. Y axis is the average prediction probability with standard errors. Prediction probability represents the probability predicted by our models for a sequence being a target sequence of a miRNA. Ref_miTAR1_Pos and ref_miTAR2_Pos represent the prediction probability from the raw sequences. MutSeed2-6_miTAR1_Pos and MutSeed2-6_miTAR2_Pos represent the prediction probability from the altered sequence.

$$\text{Left: } e_{L7}^{3l} = P_{(X_4X_5X_6)A_7} = P_{X_4X_5X_6A_7} - P_{X_4X_5X_6}$$

$$\text{Right: } e_{L7}^{3r} = P_{A_7(X_8X_9X_{10})} = P_{A_7X_8X_9X_{10}} - P_{X_8X_9X_{10}}$$

Supplementary Figure 2. An example of the calculation for the impact of the miRNA 7th nucleotide *A* in a three-nucleotide environment. A_7 represents the 7th nucleotide A. *X* represents nucleotides that surrounds A_7. The number following *X* represents the position of the nucleotide in a miRNA. $P_{X_4X_5X_6A_7}$ represents the the prediction probability for the altered sequence at miRNA position 4, 5, 6 and 7. $P_{X_4X_5X_6}$ represents the prediction probability for the altered sequence at miRNA position 4, 5 and 6. Similar meaning for the $P_{A_7X_8X_9X_{10}}$ and $P_{X_8X_9X_{10}}$.

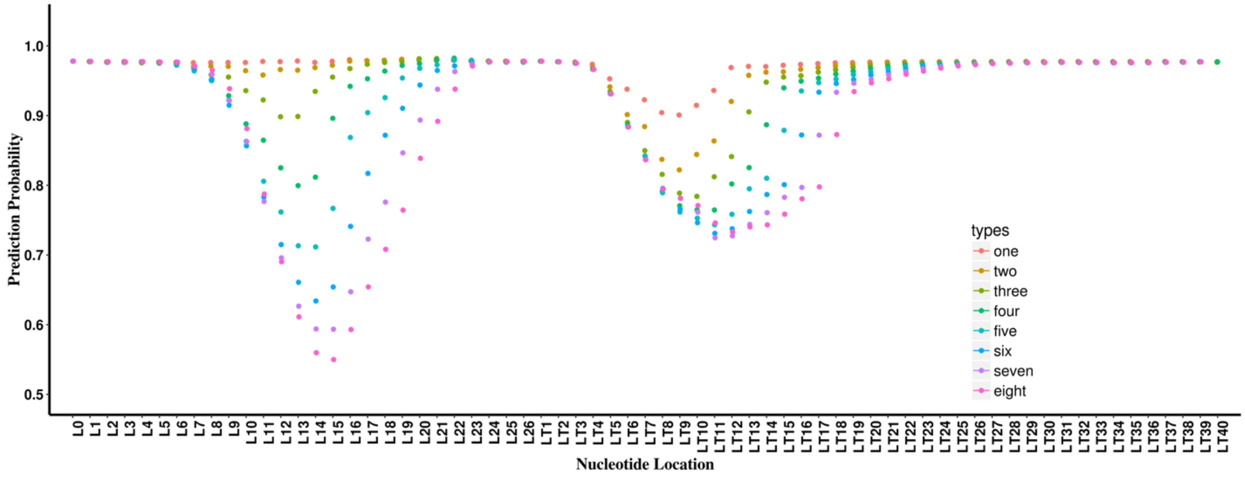

Supplementary Figure 3. The prediction probability for the miRAW positive pairs for eight types of in-silico mutagenesis analyses: one-, two-, three-, four-, five-, six-, seven-, and eight-nucleotide mutation. The labels have the same meaning as shown in Fig. 2.
